# The challenges in defining and measuring diagnostic error

**DOI:** 10.1515/dx-2014-0069

**Published:** 2015-03-12

**Authors:** Laura Zwaan, Hardeep Singh

**Affiliations:** Houston Veterans Affairs Center for Innovations in Quality, Effectiveness and Safety, Michael E. DeBakey Veterans Affairs Medical Center and the Section of Health Services Research, Department of Medicine, Baylor College of Medicine, Houston, Texas, USA

**Keywords:** clinical decision-making, cognitive errors, diagnostic error, judgment, patient safety

## Abstract

Diagnostic errors have emerged as a serious patient safety problem but they are hard to detect and complex to define. At the research summit of the 2013 Diagnostic Error in Medicine 6th International Conference, we convened a multidisciplinary expert panel to discuss challenges in defining and measuring diagnostic errors in real-world settings. In this paper, we synthesize these discussions and outline key research challenges in operationalizing the definition and measurement of diagnostic error. Some of these challenges include 1) difficulties in determining error when the disease or diagnosis is evolving over time and in different care settings, 2) accounting for a balance between underdiagnosis and overaggressive diagnostic pursuits, and 3) determining disease diagnosis likelihood and severity in hindsight. We also build on these discussions to describe how some of these challenges can be addressed while conducting research on measuring diagnostic error.

## Introduction

Diagnostic errors in medicine are relatively frequent, can have severe consequences and are only now beginning to emerge prominently in the patient safety literature [1–3]. Recent developments in the field, such as estimates of the problem and identification of downstream consequences, have successfully positioned diagnostic errors as the next challenge in patient safety [4–9]. While we have estimates of the problem in US outpatient care and in Dutch hospitals, the magnitude of harm from diagnostic error worldwide and in different health care settings remains to be accurately determined [2, 10, 11]. Studies are difficult to compare and aggregate for prevalence estimation because the definition of diagnostic error is operationalized in a variety of ways (see Table 1). In addition, measurement concepts such as diagnosis-related harm and suboptimal diagnostic processes, which are essential to diagnostic error-related work vary across studies [15]. Within the research community, there is no consensus on the definition of diagnostic error, in part due to the complexity of diagnosis.

During the 2013 Diagnostic Error in Medicine 6th International Conference in Chicago, a multidisciplinary group of 21 experts (16 physicians from diverse medical specialties, and 5 non-physician researchers) participated in a pre-conference research summit to discuss several key topics, most pressing of which was the challenge of defining diagnostic error rigorously. While we did not expect the participants to agree on one operational definition of diagnostic error, this paper aims to synthesize and build upon discussions from the summit and outlines the main challenges in operationally defining diagnostic error. We also highlight several important concepts that should be considered in research on defining and measuring diagnostic error.

## Challenge 1: Diagnosis is an evolving process

### Diseases and their manifestations often evolve over time

**Figure 1:**
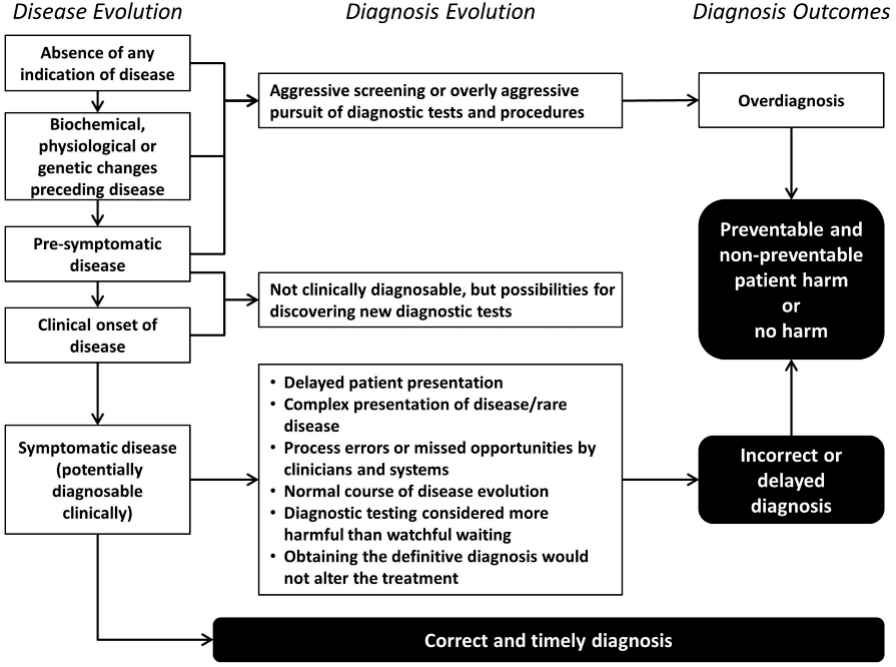
An overview of disease evolution, diagnostic process evolution and outcomes.

The discussion of defining diagnostic error should begin with the question “What is a diagnosis”? In medicine, diagnosis is the label that is attached to denote the presence of a certain disease. Physicians recognize a disease based on the pattern of symptoms, signs, test results and interpretation of all the diagnostic data. However, most diseases evolve over time and the process of evolution might be different across diseases (Figure 1). For many diseases, there is a time delay between the biochemical or physiologic onset of the disease and when a patient starts noticing symptoms (symptomatic phase). Additional time generally elapses before the symptoms are sufficiently prominent to be recognized as a disease and receive a diagnostic label. And lastly, the disease either resolves by itself, further progresses into more severe stages or is successfully treated and resolved. Sometimes the diagnostic label depends on these outcomes.

### The diagnostic process evolves over time

Information needed to diagnose a disease is often gathered by the physician in stages over time rather than all at once. During this time, the physician is obtaining additional data and considering several differential diagnoses, of which one may be the correct ultimate diagnosis. At times the available information strongly supports a wrong diagnosis, and only later does additional information emerge that allows the physician to diagnose the patient correctly. This complicates identifying diagnostic error and raises fundamental questions on error definition that need to be addressed [13]. Assume for example that a patient with cough and flu-like symptoms with temperature of 37.9°C was evaluated by physician A on day 1 and received a diagnosis of uncomplicated influenza. On day 3, the patient returned with a fever of 38.9°C and the physician B checked a chest-X-ray and diagnosed pneumonia. So now, did the disease evolve over 2 days or did physician A miss some clinical finding of pneumonia on day 1 (such as rales on chest exam)? The patient might think that physician A made a diagnostic error and perhaps even physician B. But if there was no evidence of pneumonia on day 1 and the disease just evolved, should this case be labeled as a diagnostic error?

As Figure 1 suggests, once a disease is clinically diagnosable, it may not be immediately diagnosed. Sometimes, this is because the patient delayed presenting to the physician. At other times, the diagnosis is unintentionally missed, delayed or wrong due to a process error or the clinician missing the opportunity. And lastly, this could be because immediate diagnostic testing to obtain a definitive diagnosis is not the best option, such as when diagnostic tests are invasive or harmful (watchful waiting) or when a definitive diagnosis will not alter treatment. Determination of missed, delayed or wrong diagnosis is challenging because we are often not certain exactly when the pattern of symptoms was diagnosable. This pattern is different for every disease and largely depends on the way a disease presents. In addition to this heterogeneity, studying diagnostic error retrospectively can be difficult because the physician's deliberations may not be recorded. Especially in hindsight, all of these questions are difficult to answer [16, 17].

## Challenge 2: The conundrum of over vs. underdiagnosis

Diagnostic error is mainly viewed as underdiagnosis. Historically, underdiagnosis has been a well-recognized problem and often occurs because of absence or lack of recognition of information [18, 19]. However, in contemporary medicine, newer types of diagnostic tests are very sensitive and able to detect certain conditions or abnormalities before the patient has symptoms. This increases the risk of overdiagnosis, which occurs when people without symptoms are diagnosed with a disease that ultimately will not cause them to experience symptoms or early death [20]. Overdiagnosis is more common in population-based screening, but can also occur when medical tests (laboratory and imaging) are conducted without a medical indication (e.g., “preventive” full body scans and defensive medicine). While patients are concerned about a diagnosis being missed [21] they are less aware of the consequences of overdiagnosis, which can lead to severe harm due to unnecessary treatment or unnecessary diagnostic tests. It is often not possible to determine if a specific patient was overdiagnosed [22] but recent estimates of overdiagnosed diseases (often based on comparing large groups of people who underwent early diagnostic testing to groups that did not undergo the test) are alarming [23]. Research suggests one in three breast cancers detected by population based screening is overdiagnosis [24]. Furthermore, high numbers of overdiagnosis have been found for diseases such as prostate cancer [25] and pulmonary embolism [26].

### So is overdiagnosis a diagnostic error? An example

While overdiagnosis and underdiagnosis are two different concepts and their respective studies take different methodological approaches, it is important to discuss this concept in any discussion of diagnostic error. The research summit involved discussions on the link between underdiagnosis and overdiagnosis because of their underlying relationship at the point of care, where one cannot always be comprehensively studied in isolation from the other. Take, for example, celiac disease, which is known to be underdiagnosed [27]. Getting more aggressive in diagnostic testing to reduce underdiagnosis of celiac disease in patients who present with chronic diarrhea could lead to more tests and procedures in patients who have irritable bowel syndrome. This will increase the likelihood of some of these patients carrying an incorrect diagnosis based on false-positive test results or lead to harm from such results [28]. Conversely, becoming more conservative in performing diagnostic tests or procedures in patients with chronic diarrhea might reduce overdiagnosis but will not help the underdiagnosis of celiac disease. Another reason to consider the intersection of under and over diagnosis is that consistently missing a condition might lead a clinician to become overly aggressive in their diagnostic pursuits and become more “defensive” in their approach to patient presentations associated with that condition.

Overdiagnosis associated with labeling patients with a wrong diagnosis is a type of diagnostic error, i.e., someone is told that they have a disease that they actually do not have. This is distinct from identifying true diseases that do not ultimately result in harm and from over-screening at a population level because it focuses on considerations of overdiagnosis and overtesting at an individual patient-provider level. For example, while obtaining sufficient diagnostic information to be able to correctly diagnose a disease, physicians need to strive to minimize unnecessary diagnostic testing. Research needs to take into account that one of our goals is to find the right balance to reduce harm from both under- and overdiagnosis.

To illustrate this challenge, the following case was discussed during the meeting to address this topic: “A 48-year-old woman presented to the ER with abdominal pain and one of the differential diagnoses was a small bowel obstruction. The charting very clearly reflected that this disease was considered, and documented discussions with the patient about trying a medication for constipation first and returning if that did not work. This would spare the patient a CT scan, with its risks of radiation and IV contrast, as well as costs. The medication did not work and she returned the next day; a CT scan demonstrated a small bowel obstruction due to an umbilical hernia and she was taken to the operating room to fix it”.

In this particular situation, the physician deliberately chose not to conduct a diagnostic test. If the patient ultimately only had constipation, most would judge the physician's actions as good clinical practice. However, the ultimate diagnosis of an umbilical hernia and small bowel obstruction would stimulate debate on error. The panelists' discussions if this was a diagnostic error are summarized below.

## Challenge 3: Estimating likelihood and severity in hindsight

The case discussion led experts to acknowledge that whether or not the previous case was ultimately determined to be a diagnostic error would depend on two important considerations, how urgently did the clinician need to intervene (i.e., disease severity) and what was the likelihood of the disease.

To determine whether a diagnostic error occurred in the case described above, we need to know how likely it was that the patient had a bowel obstruction or constipation. Rapid treatment of obstructed umbilical hernia is essential, so if an umbilical hernia was likely based on the clinical presentation, the majority of our panelists felt that diagnostic testing should have taken place a day earlier and would classify this as a delayed diagnosis. However, if the obstructed umbilical hernia was very unlikely based on clinical presentation and it was a lot more likely that the symptoms were due to constipation (which usually does not urge immediate treatment) watchful waiting may be considered a good clinical practice.

Diagnostic tests are often performed in cases in which a severe disease is considered even if the probability of the disease is low. Conversely, a disease that is likely but will not lead to consequences if its diagnosis is delayed might not receive immediate attention. This interaction between the likelihood of disease and urgency of treatment or intervention is constantly updated throughout the diagnostic process based on new diagnostic information that the physician acquires. For example, a red-flag symptom increases the likelihood of a severe disease and therefore the likelihood that the physician will conduct diagnostic testing. This interplay complicates the measurement of diagnostic errors, because red-flag symptoms may not always have high predictive values [29] and because this determination is different for every unique situation and involves highly subjective interpretations of likelihood and severity of diseases.

### Hindsight bias

The (estimated) likelihood and severity of diseases also influences the measurement of diagnostic error. Whether a diagnostic error occurred in an actual clinical case is determined in retrospect when more information about the correct diagnosis is available. Like all people, the reviewers who determine whether a diagnostic error occurred will be subject to hindsight bias. This means that once they know the outcome, they will think that the probability of the correct diagnosis was higher than it actually was at the time of diagnosis [16, 30, 31].

## Additional challenges for diagnostic error reduction

Given that the ultimate goal is to reduce diagnostic error in medicine and in particular diagnosis-related harm, studies need to measure harm in addition to error frequency. Preventable diagnostic errors that frequently lead to patient harm (death or disability) should be given high research and improvement priority. However, it is often not clear how to determine preventability and what types of factors to intervene upon. Untangling contributory factors for errors (cognitive factors, system factors or more than likely both) is a challenge by itself. Additionally, other contextual information such as whether the ultimate diagnosis was in the initial differential diagnosis, which health care professionals were involved in the error and the certainty of the final diagnosis are all relevant considerations in unravelling the complex interplay of factors involved in diagnostic error [32].

## Considerations to help manage challenges related to measurement of diagnostic errors

Researchers face challenges when they attempt to measure the incidence of diagnostic error because they lack an operational definition. As also shown in Figure 1, the diagnostic reasoning process evolves through a series of steps and there are many uncertainties along the way. When was the disease present? When was it diagnosable? When did the physician consider the diagnosis? Why did the physician not diagnose correctly or in a timely fashion?

So, how do we take some of these uncertainties into account when studying diagnostic error? We suggest the following considerations when conducting a study on measurement of diagnostic error.

### Determine which concepts and definitions are right for your study

It is important to consider which concepts (e.g., diagnostic error, harm from diagnostic adverse events, missed opportunities) and/or definitions will fit best with the research aims [2, 12–14]. A broad definition of diagnostic error that includes all delayed, wrong and missed diagnoses might tend to overestimate the size of the problem because many diseases take time to make it to a stage when they are ready to be clinically diagnosed (as we discuss previously in the evolving diagnosis section). A more precise definition which includes only missed, delayed and wrong diagnosis that were caused by human and/or system factors will provide insight into errors that could be prevented and therefore highlight opportunities for error prevention [33]. Furthermore, it may be most important to prevent patient harm, and therefore studies could also focus on diagnostic errors in specific disease conditions that are more likely to cause harm [8].

While the approaches mentioned herein each have advantages and disadvantages, it might be best to operationalize the definitions of errors in a standardized fashion so that measurement related studies are comparable. We ultimately need a uniform conceptual foundation that provides a common shared understanding of diagnostic error and takes into account different unique perspectives.

Operationalizing the definition of diagnostic errors differently for each disease condition might be one way to study them. When specific diseases are studied with detail and rigor, the complex concepts about missed, delayed or wrong diagnosis discussed herein might be more easily operationalized. For example, one could decide that for patients newly diagnosed with lung cancer, if in hindsight there was no follow-up performed within 7 days of an abnormal imaging report suggestive of cancer, it would be considered a diagnostic error [34]. However, not every research aim allows disease-specific operationalization. If the study aims require a diverse sample of diseases to obtain insight on incidence rates of different types of diagnostic errors, a less precise operationalization is inevitable.

### Account for uncertainty in measurement

The current practice in research is that the presence of a diagnostic error is determined on a dichotomous scale and reviewers determine whether diagnostic error is present or absent. Given the complexities and uncertainties discussed in this paper, it is clear why in everyday clinical practice this is not so black and white. One strategy to take these uncertainties into account is to determine the occurrence of diagnostic error on a scale rather than present or absent. In research on adverse events in medicine, a 6-point scale is commonly used to determine the extent to which health care-related harm was caused by the health care system as well as to determine the extent of the preventability of harm. The scale used in these studies includes the following six levels: 1. (Virtually) no evidence, 2. Slight to modest evidence, 3. Not likely ( < 50/50, but “close call”) 4. More likely (more than 50/50, but “close call”) 5. Moderate to strong evidence 6. (Virtually) certain evidence [35–37]. Another option is to use a scale for degree of agreement with a statement about the presence of diagnostic error (strongly agree to strongly disagree). Such scales could help identify not only the obvious error cases but also help obtain insights about the cases where there is uncertainty about whether a diagnostic error occurred.

Further research is needed to determine the optimal number of opinions to determine whether an error occurred. For example, it has been suggested that more reviewers could lead to better estimation of the incidence of adverse events through medical record reviews [38]. Conversely, another study showed that having two reviewers did not outperform having one reviewer [39]. Research on these psychometric properties could enable development of more reliable and valid measurement techniques in the future.

### Consider triangulation of data sources and research methods

The types of medical errors that are identified largely depends on the data source and research methods used [40, 41]. This is also relevant for diagnostic error research. For instance, identifying errors through chart reviews will identify different types of diagnostic errors than the errors reported by patients. Furthermore, prospective methods will find different types of diagnostic errors than retrospective methods. Each of these will provide a slightly different viewpoint on error measurement. To develop a more comprehensive overview of the problem, triangulation (i.e., using a combination of data sources and research methods) is important. Evaluating the problem using different perspectives and methods will allow a more reliable estimate of the problem.

## Conclusions

We build upon discussions of the 2013 Diagnostic Error in Medicine Conference research summit and identify several key challenges of defining and measuring diagnostic error in medicine. Some of these challenges include the evolving nature of the disease, the evolving nature of the diagnostic process, what is a diagnostic error, the subjectivity when determining error in hindsight, not always having a concrete black and white answer on whether error is present or absent, and the need to balance the risks of underdiagnosis and over-aggressive pursuit of a diagnosis. We recommend that researchers consider strategies to address and overcome these challenges while conducting studies on measuring diagnostic error. Some of these strategies could include operationalizing the definitions of errors in a standardized fashion so that measurement-related studies are comparable especially across similar disease conditions, measuring diagnostic error on a scale rather than dichotomously and using triangulation of data sources and research methods to support more rigorous measurements.

## Figures and Tables

**Table 1:** Overview diagnostic error definitions [12-14].

Term	Definition	Defined by
Diagnostic error	A diagnosis that was unintentionally delayed (sufficient information was available earlier), wrong (another diagnosis was made before the correct one), or missed (no diagnosis was ever made), as judged from the eventual appreciation of more definitive information.	Graber et al. [12]
Diagnostic error	Missed opportunities to make a correct or timely diagnosis based on the available evidence, regardless of patient harm.	Singh [13]
Diagnosis error	Any mistake or failure in the diagnostic process leading to a misdiagnosis, a missed diagnosis, or a delayed diagnosis. This could include any failure in timely access to care; elicitation or interpretation of symptoms, signs, or laboratory results; formulation and weighing of differential diagnosis; and timely follow-up and specialty referral or evaluation.	Schiff et al. [14]
